# Treatment of central line-associated bloodstream infections

**DOI:** 10.1186/s13054-018-2249-9

**Published:** 2018-11-16

**Authors:** Jérémy Guenezan, Bertrand Drugeon, Nicolas Marjanovic, Olivier Mimoz

**Affiliations:** 1 Centre Hospitalier Universitaire de Poitiers, Service des Urgences Adultes & SAMU 86-Centre 15, 1 Rue de la Milétrie, 86021 Poitiers Cedex, France; 2 Université de Poitiers, UFR de Médecine Pharmacie, Poitiers, France; 3 INSERM, U1070, Pharmacologie des Agents anti-Infectieux, Poitiers, France

**Keywords:** Central venous catheter, Bloodstream infection, Prevention, Antibiotics

Central venous catheters (CVCs) are among the most frequently used medical devices in critically ill patients [1]. Unfortunately, complications are not uncommon despite the many improvements in caregiver behaviour observed over recent decades. Central line-associated bloodstream infection (CLABSI) is probably the most feared complication [2]. It is defined as a primary laboratory-confirmed bloodstream infection in a patient with a central line at the time of, or within 24-h prior to, the onset of symptoms, in cases where the cultured organism is not related to an infection from another site. The central line should be in place for > 2 calendar days on the date of the event, with the day of device placement being day 1. Tens of thousands of patients continue to experience CLABSIs each year in the US, resulting in thousands of deaths each year and billions of dollars in added costs to the US healthcare system [3].

Of paramount importance, CLABSI prevention depends on evidence-based measures. The most effective of these are hand hygiene before catheter insertion and maintenance, full-barrier precautions during catheter insertion, 2% alcoholic chlorhexidine use for skin preparation, femoral vein avoidance whenever possible and prompt removal of useless CVCs [4–6]. The use of care protocols and the training of caregivers must also be considered. Implementation of these bundled measures has been shown in high-quality studies to reduce CLABSI incidence by 50% [7].

On the other hand, the principles of CLABSI treatment are not as well established and are mainly based on expert opinions and the results of old cohort studies [2]. Source control and intravenous administration of high doses of bactericidal agents are the most commonly cited measures.

Source control consists of prompt removal of the catheter when clinical suspicion is high, at times even before the CLABSI diagnosis is proven. Yet, catheter salvage with antibiotic lock and systemic antibiotic treatment are not recommended for critically ill patients. Salvage therapy is limited to cases where the CVC cannot be removed, in the absence of septic shock, and for a period of at least 4 weeks, with low success rate.

Empirical treatment should be initiated promptly, before confirmation of the diagnosis, after appropriate cultures have been obtained. The choice is based on host characteristics, known or suspected colonization with resistant microorganisms and local epidemiology and susceptibility patterns [8]. In general, reasonable coverage is required for common Gram-positive and Gram-negative organisms. Parenteral vancomycin (or daptomycin in patients with acute renal failure or when organisms have reduced susceptibility to vancomycin) is the cornerstone of an empirical regime in settings with a high prevalence of methicillin-resistant Staphylococcus aureus (MRSA) [9]. Otherwise, an anti-staphylococcal penicillin or a first-generation cephalosporin is enough [10]. Antibiotics active against Gram-negative bacilli include β-lactam/β-lactamase inhibitor combinations, fourth-generation cephalosporins and carbapenems alone or in combination with an aminoglycoside [11, 12]. Agents against *Pseudomonas aeruginosa* are required in cases of neutropaenia, severity of disease or increased probability or known prior colonization [8]. New anti-Gram negative antibiotics are indicated for infections due to multi-drug resistant Gram-negative organisms [13].

Empiric coverage for candidaemia should be considered if multiple sites are colonized with *Candida* or for patients with bone marrow or organ transplants, hematologic malignancy, femoral catheterization or when patients are receiving total parenteral nutrition or prolonged administration of broad-spectrum antibiotics [2, 8]. Parenteral echinocandins are recommended when resistance to azoles is suspected (prior exposure to azoles in the previous 3 months or high prevalence of common non-*albicans Candida* such as *Candida glabrata* or *Candida krusei*) [8, 12]. Otherwise, intravenous fluconazole is enough.

Once antimicrobial susceptibility patterns are available, de-escalation to appropriate therapy with the narrowest spectrum possible is recommended [14]. The duration of treatment depends on the organism involved and the presence of complications or not, and varies between 7 days to 6 weeks.

For uncomplicated infection (i.e., not associated with suppurative thrombosis, endocarditis or metastatic infection) with septic manifestations resolving within 72 h of catheter removal, intravenous antimicrobial therapy is recommended for the following duration based on the organism isolated (Fig. 1):

- *Staphylococcus aureus*: 14 days
- Coagulase-negative staphylococci: 7 days
- Enterococci and Gram-negative bacilli: 10 to 14 days
- *Candida*: 14 days

**Fig. 1:**
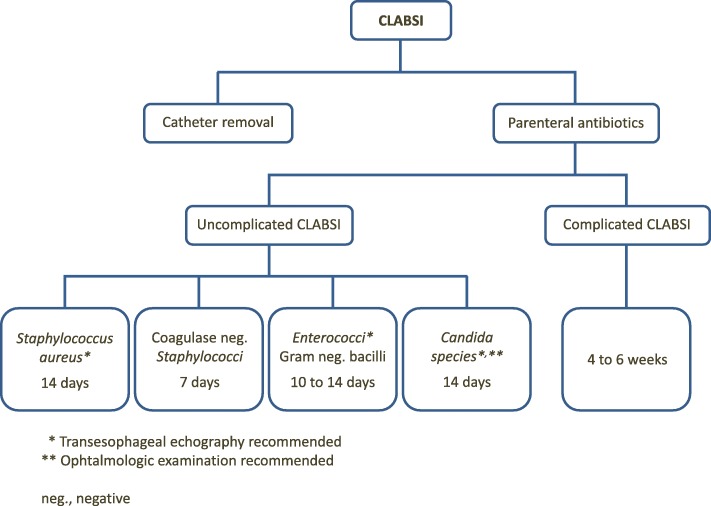
Approach to the treatment of a patient with central line-associated bloodstream infection (*CLABSI*)

Recently, a retrospective cohort study of 366 uncomplicated CLBSI cases added new scientific evidence on the subject [15]. Duration of antimicrobial treatment lower than 15 days was associated with earlier mortality or recurrence, suggesting that antibiotic treatment should be continued for at least 2 weeks for all microorganisms. Unfortunately, the study was not powerful enough to compare outcomes amongst individual classes of causative organisms. The authors concluded that further evidence is required before amending the guidelines.

Transoesophageal echography should be performed in patients with *S. aureus*, enterococci and *Candida* infections so as to rule out complicated forms of CLBSI [2]. Similarly, an ophthalmological examination is recommended in candidaemic patients [2]. Indeed, the duration of parenteral antibiotic therapy should be longer in the case of complicated CLABSIs, ranging from 4 to 6 weeks, even though there is little scientific evidence to support these recommendations [14]. Suppurative thrombophlebitis may also require anticoagulation and sometimes surgery. Infectious endocarditis and osteomyelitis may require surgical treatment.

## Conclusion

The treatment of CLABSIs in 2018 is still based on expert opinions and cohort studies rather than robust scientific evidence. Such infections continue to increase complications and mortality and are quite costly. Large-scale studies are warranted to further clarify the nature and duration of treatment. In the meantime, physicians are encouraged to use algorithms such as the one proposed here (Fig. 1).

## Abbreviations

CLABSI: Central line-associated bloodstream infection
CVC: Central venous catheter
